# Homing trajectories and initial orientation in a Neotropical territorial frog, *Allobates femoralis* (Dendrobatidae)

**DOI:** 10.1186/1742-9994-11-29

**Published:** 2014-03-25

**Authors:** Andrius Pašukonis, Matthias-Claudio Loretto, Lukas Landler, Max Ringler, Walter Hödl

**Affiliations:** 1 Department of Cognitive Biology, University of Vienna, Althanstrasse 14, 1090 Vienna, Austria; 2 Department of Biological Sciences, Virginia Tech, Blacksburg, VA 24061, USA; 3 Department of Integrative Zoology, University of Vienna, Althanstrasse 14, 1090 Vienna, Austria; 4 Department of Tropical Ecology and Animal Biodiversity, University of Vienna, Rennweg 14, 1030 Vienna, Austria

**Keywords:** Homing, Orientation, Telemetry, Dendrobatidae, *Allobates femoralis*

## Abstract

**Introduction:**

The ability to relocate home or breeding sites after experimental removal has been observed in several amphibians and the sensory basis of this behavior has been studied in some temperate-region species. However, the actual return trajectories have rarely been quantified in these studies and it remains unknown how different cues guide the homing behavior. Dendrobatidae (dart-poison frogs) exhibit some of the most complex spatial behaviors among amphibians, such as territoriality and tadpole transport. Recent data showed that *Allobates femoralis*, a frog with paternal tadpole transport, successfully returns to the home territories after experimental translocations of up to 400 m. In the present study, we used harmonic direction finding to obtain homing trajectories. Additionally, we quantified the initial orientation of individuals, translocated 10 m to 105 m, in an arena assay.

**Results:**

Tracking experiments revealed that homing trajectories are characterized by long periods of immobility (up to several days) and short periods (several hours) of rapid movement, closely fitting a straight line towards the home territory. In the arena assay, the frogs showed significant homeward orientation for translocation distances of 35 m to 70 m but not for longer and shorter distances.

**Conclusions:**

Our results describe a very accurate homing behavior in male *A. femoralis*. The straightness of trajectories and initial homeward orientation suggest integration of learned landmarks providing a map position for translocated individuals. Future research should focus on the role of learning in homing behavior and the exact nature of cues being used.

## Introduction

The ability to quantify individual movement patterns using modern telemetry techniques has been crucial in understanding animal orientation. Homing ability and trajectories following translocations from home or breeding sites have been widely used to study the orientation mechanisms involved (e.g., homing pigeons [1], desert ants [2]).

Directed, relatively long distance movements, such as mass spring migration to breeding ponds in temperate-region amphibians, has historically drawn much attention to amphibian orientation ([3,4], reviewed in [5,6]). A tendency to move towards and to relocate home or breeding sites after experimental removal has been observed in many amphibians (e.g., *Anaxyrus terrestris* (Bufonidae) [7], *Lithobates pipiens* (Ranidae) [8], *Pseudacris regilla* (Hylidae) [9], *Allobates femoralis* (Dendrobatidae) [10], *Taricha rivularis* (Salamandridae) [11]). Several sensory modalities have been implicated in this ability, most commonly olfaction [12-14], magneto-reception [15,16], and a celestial compass [5,17]. Often, integration and compensation of different modalities depending on cue availability have been suggested (reviewed in [6,18]). Even though the importance of certain sensory modalities has been revealed for some temperate-region amphibians, the exact homing trajectories have not been quantified (but see [3,4,18,19]). Thus it remains unknown how exactly these cues guide the behavior.

Traditionally, orientation mechanisms in amphibians have been studied by observing initial orientation over a few meters in arena setups or over longer distances using drift fences and pit-fall traps. In an attempt to track individuals, several authors have attached trailing devices to the backs of larger anurans, i.e. a thread bobbin, which releases a thread as the animal moves [8,13,20,21]. However, this tracking method hinders animal movements, often results in injuries or a complete inhibition of homing [8,22], and is limited to large species. The miniaturization of radio transmitters has strongly expanded the range of species suitable for telemetry, including many amphibians [23,24]. In parallel, harmonic radar [25] and harmonic direction-finding techniques [26] were developed, allowing to track even some of the smallest amphibians. Unfortunately, limited detection range and problematic external attachment on amphibians have resulted in predominantly methodological studies (e.g., [27-29], but see [30,31]). Telemetry techniques have rarely been used to gain new insights into amphibian orientation.

Orientation has mostly been studied in nocturnal temperate-region anurans, especially bufonids, but it is in tropical amphibians that we find some of the most complex spatial behaviors. Dart-poison frogs (Dendrobatidae) are a group of diurnal Neotropical frogs characterized by territoriality and parental care, which includes tadpole transport [32]. Despite the fact that dendrobatid parental care has been investigated in great detail [32-34], very little is known about the related spatial behaviors. *Allobates femoralis* is a small dendrobatid frog common throughout the Amazon basin and the Guiana Shield [35]. At the onset of the rainy season, males establish territories, which are vocally advertised and defended for up to several months [36,37]. Courtship, mating and oviposition take place in the leaf-litter within the male’s territory. Tadpoles are later transported to widely dispersed aquatic deposition sites, such as temporary pools in flooded areas or holes in fallen trees, as far as 185 m away from the territory [38]. Recent data have shown that experimentally translocated territorial males can return from up to 400 m with increased homing success rates under 200 m [10].

In the present study we further describe the homing behavior of male *A. femoralis* with a specific focus on the actual movement patterns during return to their home territory. We used telemetry with miniature passive transponders to quantify the homing trajectories of territorial males, displaced over 50 m. Additionally, we translocated males from their home territories into a circular arena within their natural habitat and quantified the initial movements after release.

## Results

### Telemetry experiment

**Figure 1:**
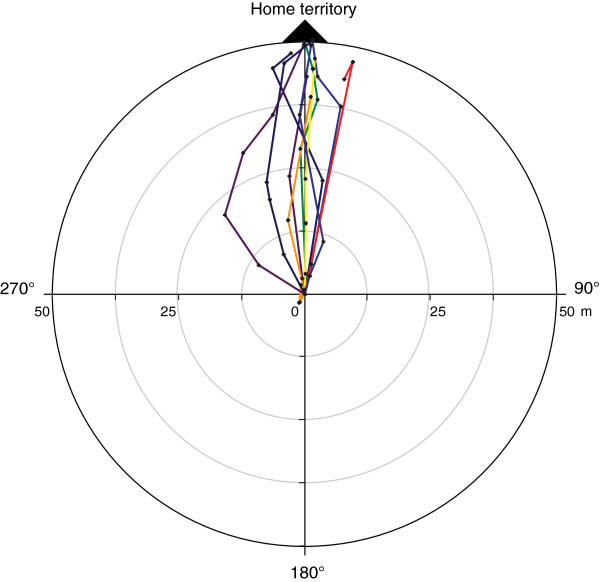
**Homing trajectories circular.** Circular plot showing the homing trajectories of ten territorial males that were translocated over 50 m in the telemetry trials. For better visualization, all trajectories were normalized to a single starting point and axis. Each line represents a different individual. Points mark en route locations connected by linear interpolation.

Fifteen out of 17 male frogs equipped with transponders continued to show territorial behavior. Out of 15 individuals translocated for 50 m, 12 successfully returned to their home territories. Three individuals did not move from their release sites for three to four days, after which we removed their tags and returned them to their home territory to avoid any long-term effect on individuals’ health and behavior. Two individuals lost the reflector antenna and could not be located en route but were recovered back in their home territories. Ten individuals were successfully tracked en route. On average the trajectory straightness coefficient was very high (Mean_SC_ = 0.93, SD = 0.075) and the inferred homing trajectories were closely fitting a straight homewards line (Figure 1). Translocated individuals showed a significant initial homeward orientation (Mean = 344.5°, 95% CI = 304.7° – 24.3°, Rayleigh-test p = 0.01, n = 8).

Total return time ranged from 4 h to 102 h (Mean = 37.82 h, SD = 29.49). During their diurnal activity period, individuals spent significantly more time immobile than moving (median_moving_ = 4.88 h, Q1–Q3_moving_ = 4–8.06 h, median_stationary_ = 14.88 h, Q1–Q3_stationary_ = 4.38–23.13 h, Z = -1.97, Wilcoxon p = 0.05, n = 10). As seen from the quartile values, there was relatively little variation in the active movement time necessary for frogs to return to their home territory but a much greater variation in the time they spent immobile.

### Arena assay

**Figure 2:**
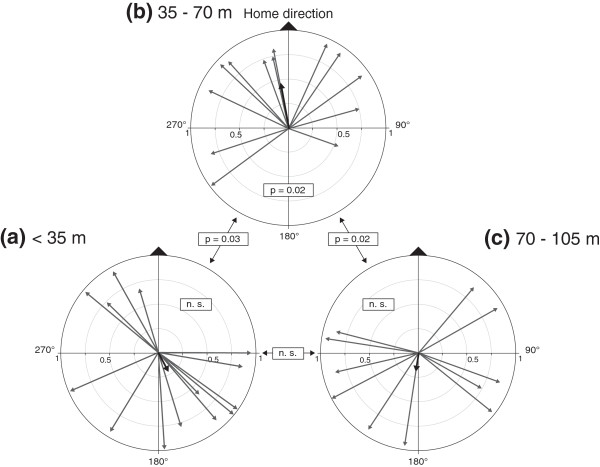
**Arena orientation.** Circular vector plots representing initial orientation over one meter of territorial individuals translocated from close-range, < 35 m **(a)**, mid-range, 35 – 70 m **(b)**, or far-range, 70 – 105 m **(c)**. Each vector represents an individual. Vector length represents the path straightness in the arena. Vector direction represents the bearing from the arena center to the crossing point of a one-meter radius circle. Reported significance levels were obtained by second order Hotelling’s circlular test for unimodal distribution and by a Hotelling’s two-sample test for the comparisons between distributions.

A second order circular tests for unimodal distribution revealed a significant homeward orientation for the individuals from the mid-range (n = 13, Mean vector = 345.1°, 95% CI = 275.7° – 49.1°, Hotelling’s F = 5.49, p = 0.02) but not for the close-range (n = 14, Hotelling’s p > 0.1) and far-range samples (n = 11, Hotelling’s p > 0.1) (Figure 2). Confirming the between-group difference, there was a significant difference in distribution between the mid-range and the close-range (Hotelling’s two sample F = 4.06, p = 0.03) as well as mid-range and far-range samples (Hotelling’s two sample F = 4.64, p = 0.02) but not between the close and far-range samples (Hotelling’s p > 0.1). Overall, we could not unambiguously confirm a successful homing after the trial for six individuals (three for the close-range and three for the-far range).

## Discussion

**Figure 3:**
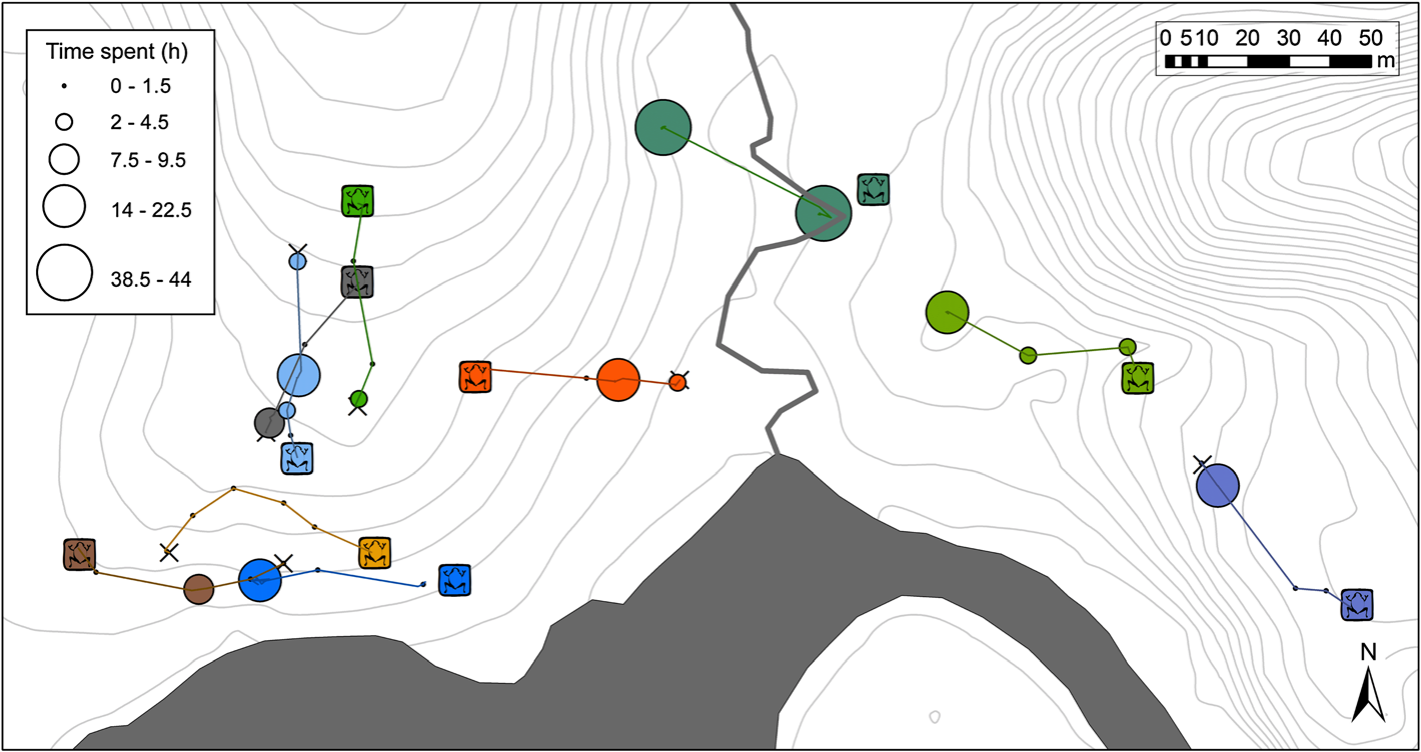
**Trajectory map.** Map of the study area showing homing trajectories and temporal distribution of ten territorial males, each individual represented by a different color. Square frog symbols indicate the home territories; x-symbols show release points; circles show en route locations connected with linear interpolation lines. The size of each circle is proportional to the time spent at each location. Contour lines (1 m) are in light gray, creeks and Arataye River in dark gray.

Our tracking experiment revealed that homing trajectories of translocated male *A. femoralis* are characterized by rapid movements, closely fitting a straight line towards the home territory, with occasional long periods of immobility lasting up to several days (Figure 3). We did not observe any search patterns and the initial movements over several meters after release were already oriented homewards. Detailed analysis of the very first movements within one meter in an arena setup revealed significant orientation for translocation distances of 35 m to 70 m but not for longer and shorter distances.

The most intriguing finding is the accuracy of the observed homing trajectories. The straightness of trajectories over similar distances is more comparable with path integration based homing in desert ants (e.g., [2]) or direct guidance in social insects (e.g., [39]) rather than any homing trajectories described in amphibians [8,40]. However, experimental translocation from territories excludes the possibility of path integration in our study. Direct guidance (also termed beaconing) requires direct sensory contact to the goal. We can exclude direct visual contact with the goal in the dense rainforest. The role of olfaction in orientation has been demonstrated for several anuran species, but in these cases animals were orienting in an open landscape to a large goal such as a breeding pond [12-14,20]. It is difficult to imagine how olfactory guidance would explain such an accurate homing to small terrestrial territories in a forest understory with hardly any stable winds. Alternatively, it has been suggested that some animals could form ‘olfactory maps’, which allow them to navigate an area, but the empirical evidence is equivocal [41]. Magnetic compass and magnetic maps have been implicated in long distance homing of several species [42], including urodele amphibians [15,16], but the shallow magnetic gradients of the earth’s magnetic field could not account for the observed homing precision at these relatively short distances [43]. Acoustic orientation has been well documented for male *A. femoralis* in a territorial defense context [44-46] and females most likely use males’ advertisement calls as beacons to find territory owners over several tens of meters [47]. We speculate that translocated individuals could use other territorial males as acoustic beacons or even integrate their calling positions and identities into a full acoustic map of the area. To the best of our knowledge, the potential existence of such orientation mechanism has not yet been investigated. Broad acoustic gradients such as provided by a nearby river could be used in addition. The function of broad acoustic gradients in homing has been often discussed in anuran orientation, usually in the context of a breeding chorus (e.g., [7,14,48]), but there is little evidence of acoustics being a primary cue in anuran homing.

Other types of landmark-based orientation are also possible. However, the lack of any observable search patterns and strong initial orientation suggest, that if spatial learning is involved, landmarks may be integrated on an internal map and not simply used as intermediate beacons. Translocation distances of 50 m are well inside the potential range explored by most individuals during tadpole transport [38] or juvenile dispersal (M. Ringler, R. Mangione, A. Pašukonis, E. Ringler, unpublished data) and spatial information could be acquired during these movements. Homing success in *A. femoralis* is very high up to 200 m [10], which corresponds to the maximum distances covered by tadpole transporting adults, and further suggests the role of familiarity with the area in homing behavior. More generally, many dendrobatid frogs are likely to rely on spatial learning for finding small and widely dispersed aquatic tadpole deposition sites in complex environment but research in this area is lacking.

The long immobility periods shown by most individuals before moving could be related to the time necessary to accumulate or to perceive temporally varying orientation cues, such as conspecific vocalizations. We did not observe any clear relation between frogs’ activity during homing and the time of the day or the amount of precipitation. We do not think that catching or tag attachment induced stress caused these latencies. Our experience from a long-term recapture study of *A. femoralis* has shown that most males resume their previous activities such as calling or courtship after being caught or even toe-clipped ([49] and personal obs. by A. Pašukonis). Further, the average return time from 50 m for non-tagged animals (2 days, [10]) is similar to the one observed for tagged individuals in this study (38 h). Of course, it is possible that the removal from a familiar location results in stress induced passiveness and immobility. However, we regularly observed translocated frogs waiting for hours on exposed, slightly elevated structures at the release site, which is an indication of an alert state rather than passiveness.

Initial movements of frogs translocated into the arena for distances comparable to the tracking experiment (35–70 m) support the assumption that *A. femoralis* perceives some cues directly at the release site. However, the overall results are equivocal, as the individuals with territories closer than 35 m and further than 70 m to the arena did not show significant homeward orientation. A strong motivation to escape from the arena might override the immediate homing motivation. More specifically, frogs displaced over very short distances might have had less motivation to orient towards their territory before leaving the arena. On the other hand, individuals coming from further than 70 m might have not been able to perceive the orientation cue within the given trial time. In the arena, most individuals moved within the first 90 min and the remaining few were excluded from the trial, while after the release at natural sites in the tracking experiment, some frogs remained stationary for more than 24 h before initiating their movements. This indicates, that if the orientation cues are only temporally available or need to be accumulated for correct orientation, the arena setup was not suitable to test for this ability. It is important to note, that even the males that were displaced to the arena from longer distance returned to their home territories. Additional tracking of individuals after testing them in the arena would be necessary to understand how the direction choice in the arena relates to the overall homing motivation and trajectories.

To the best of our knowledge, this is the first study quantifying how territorial frogs return after experimental removals. It is also one of few studies addressing amphibian orientation by quantifying full homing trajectories. Even though the HDF method has its clear limitations such as short detection range (10–30 m) and no possibility to identify individuals based on the signal, we demonstrate that it can be a useful tool for understanding movement patterns of small animals in complex environments, such as the tropical rainforest. Several authors have reported problems, such as altered behavior and injuries, from external attachment methods on anurans [28,31,50]. Our attachment method was generally suitable for a short-term (up to five days) tracking but could be problematic for longer-term studies. Frogs exhibited all their natural reproductive behaviors such as calling, territorial defense, courtship, and on one occasion even tadpole transport while having a reflector attached. However, on some occasions, we observed skin abrasions around the waist after removing the tag. Observed injuries healed in a few days and at least in some cases we know that males continued to defend territories for weeks and months following the tracking.

## Conclusions

Overall, our results underline the importance of quantifying the individual trajectories after experimental displacements in understanding amphibian orientation. Taken together with measures of initial homeward orientation, they reveal a very accurate homing behavior in *A. femoralis*. The straightness of trajectories and initial homeward orientation suggest an integration of learned landmarks providing a map position for translocated individuals. We suggest that spatial knowledge acquired during juvenile dispersal or tadpole transport mediates this behavior but the actual cues being used remain unknown. Future research should focus on the importance of familiarity with an area for successful homing and the nature of sensory cues being used.

## Materials and methods

### Study animals and area

*Allobates femoralis* is a small (snout-urostyle length approximately 25–30 mm) territorial dendrobatid frog. Playback of an advertisement call of a simulated intruder reliably elicits antiphonal calling or direct phonotactic approach by the resident male [44]. Individual frogs can be identified and recognized by their unique ventral coloration patterns [49].

The study was carried out within one reproductive season of *A. femoralis* between 19 January 2013 and 30 March 2013. Frogs were sampled from a single population in an area of approximately 3 ha near the field camp ‘Saut Pararé’ (4°02’ N, 52°41’ W, WGS84) in the nature reserve ‘Les Nouragues’, French Guiana. The study area mainly consists of primary lowland rainforest bordering the ‘Arataye’ river to the south.

### Telemetry experiment

We used the harmonic direction-finding (HDF hereafter) telemetry technique to obtain homing trajectories of experimentally translocated territorial males. The HDF system consists of a directional transceiver and a passive reflector. The transceiver emits and recaptures a radio signal which gets reflected from the tag attached to an animal, thereby providing directional information (for more details see [26,29]). The miniature size of the reflector tags allows this technique to be used on smaller animals than permitted by conventional active radio tracking. We used a commercially available transceiver (R8, RECCO® Rescue System, Lidingö, Sweden) with reflector tags consisting of a Schottky diode soldered between two antennas. Antennas were made of 40 μm steel strands forming a 2 cm by 10 cm T-shaped dipole with the braze point sealed in a heat-shrink tubing. We attached the reflectors to the frogs using a waistband made of 1 mm diameter silicon tubing similarly to Gourret and Schwarzkopf [27]. The short part of the T-shaped antenna was secured inside of the tube and the waistband was fixed with a cotton thread going through the inside of the tubing. The tag together with the attachment made up for less than 5% of frogs’ body weight (range_frog weight_ = 1.4 – 2.3 g, max_tag weight_ = 0.07 g).

The telemetry experiment took place from 21 January 2013 to 15 March 2013. During this period, 18 territorial males were captured and equipped with reflector tags. Calling males were detected and identified as territorial if they showed stereotypical territorial defense behavior (calling and phonotactic approach), which was elicited by broadcasting conspecific advertisement calls, simulating an intruder. Frogs were captured with transparent airtight plastic bags, photographed for identification, and their precise capture positions were recorded with the mobile GIS software ArcPad™ 10.0 (ESRI, Redlands, CA, USA) on pocket computers (MobileMapper™ 10, SpectraPrecision, Westminster, CO, USA) using a detailed background map based on a grid of reference points and natural structures. Each frog was equipped with a reflector and immediately released at its initial capture position, where it was observed for a 24 h period to confirm normal territorial behavior with the reflector. Each frog was located at least once during this period and confirmed to behave territorially if it showed any of the following behaviors: calling, phonotactic approach of a simulated intruder, courtship. Two males were excluded at this stage and their tags were removed because no territorial behavior was observed, and another individual lost the waistband and could not be located again.

When normal territorial behavior with the tag was confirmed, each frog was captured with an airtight plastic bag, placed in an opaque container and translocated 50 m away from the home territory (n = 15). Each animal was designated to one of five displacement directions (N, E, S, W and NW), avoiding terrain and vegetation where tracking might have been impossible. The release points were located and marked using the same detailed background map on a pocket computer. *Allobates femoralis*, like most dendrobatid frogs, is diurnal and does not move during the dark hours, which was confirmed by preliminary observations and during this study. Therefore, individuals were tracked during the daylight hours (07 00 h to 19 00 h) until they returned to their territories. Locations were visited approximately every hour with occasional shorter intervals when the frog was found actively moving. Longer intervals were sometimes forced by bad weather conditions and/or additional time taken to relocate the frog.

To locate the frogs, we followed the increasing amplitude of the reflected signal until visually spotting an individual. In cases of poor visibility or if an individual was hiding in the leaf litter, we narrowed the signal to less than one meter. If an individual remained stationary and hidden for longer periods, we carefully uncovered the frog at least once a day to make sure that the tag had not fallen off and that the individual had no injuries. Occasionally, the tag had twisted to the side or underneath the frog, in which case the frog was carefully manipulated by pulling the antenna to reposition the tag. These manipulations never took more than a minute and frogs never moved more than a meter as a result of them. Because the harmonic signal does not carry an individual signature and handling would be necessary for identification, we never translocated more than one frog at a time in the same area.

### Trajectory analysis

Initial visualization, extraction of coordinates and distance measurements were done in the GIS software ArcGIS™ 10 (ESRI, Redlands, CA, USA). The geographic coordinates of all locations were projected (UTM, zone 22 N, WGS84) and extracted as X- and Y-coordinates in meters. We grouped points spaced less than two meters apart to a single position, as they might have fallen within the measurement error of the exact position in the field.

We calculated a straightness coefficient (SC) for the path between the release site and the home territory as the ratio between the straight-line distance and the actual path distance, with a ratio of one indicating a perfectly straight path. To test for initial homeward orientation, we considered the bearing between the release point and the first position further than two meters away from the release point (n = 8, mean_distance to__first position_ = 6.6 m, SD = 4.2). Significant homeward orientation was tested using Rayleigh’s test for unimodal distribution. Two individuals were excluded from this analysis because the first recovery positions were too far (> 20 m) from the release point to be seen representative for initial orientation. The Rayleigh test was performed with a circular statistics program Oriana 4.02 (Kovach Computing Services, Pentraeth, Wales, UK).

### Return time analysis

To analyze return times, we considered diurnal activity hours from 7 00 h to 19 00 h. We further split the total return time of each individual into time actively moving and time stationary. Any time interval where an individual moved further than two meters between two consecutive relocalizations was considered as time active. We compared the two activity modes using the Wilcoxon Signed-Rank test for comparison of paired samples using the R Stats package 3.0.1 [51].

### Arena assay

We further assessed the initial orientation ability of male *A. femoralis* in an experimental arena setup. A circular arena with a diameter of 240 cm and 40 cm height was set up at an arbitrarily chosen location in the natural habitat. The walls of the arena where made out of blue plastic tarpaulin and supported by fixed poles, while the floor was covered with an off-white, thick, plastic sheet, marked with a grid as a reference for position measurements.

To test for initial orientation after experimental translocation, territorial males were caught in their territories and released in the arena, following the same catching and translocation protocol as in the telemetry trials. Single individuals were placed under a release device in the center of the arena and left to habituate for 15 min.

The release device consisted of two upturned plastic flowerpots, stacked one over the other. After the outer part was lifted with a string, the frog could leave the inner part to all directions through cutout exit holes. This release design was necessary because some individuals showed immediate escape behavior when left exposed with no cover. Trials were filmed for 90 min without experimenter presence using a wide angle video camera (GoPro™ HD HERO2, Woodman Labs Inc., San Mateo, CA, USA) on a tripod. After leaving the release device and reaching the edge of the arena, frogs usually circled and eventually climbed over the wall. Individuals were left to home back to their territories. Frogs that did not exit the release device within 90 min were returned to their home territories and excluded from the analysis.

Fifty-six arena trials were conducted from 19 January 2013 to 30 March 2013. Nine trials were excluded for technical problems (n = 3) or because frogs did not leave the release device within 90 min (n = 6). Comparisons of individual photographs revealed that nine individuals where tested twice and in such cases only the first trial was included in the analysis.

Initially, we translocated frogs that had their territories within 30–70 m around the arena to enable a direct comparison with the homing trajectories of the frogs that were used in the tracking experiment where they were translocated over distances of 50 m. Subsequently, we included frogs closer (5–30 m) and further away (70–105 m) from the arena to assess the effects of translocation distance on the accuracy of initial orientation.

### Initial orientation analysis

**Figure 4:**
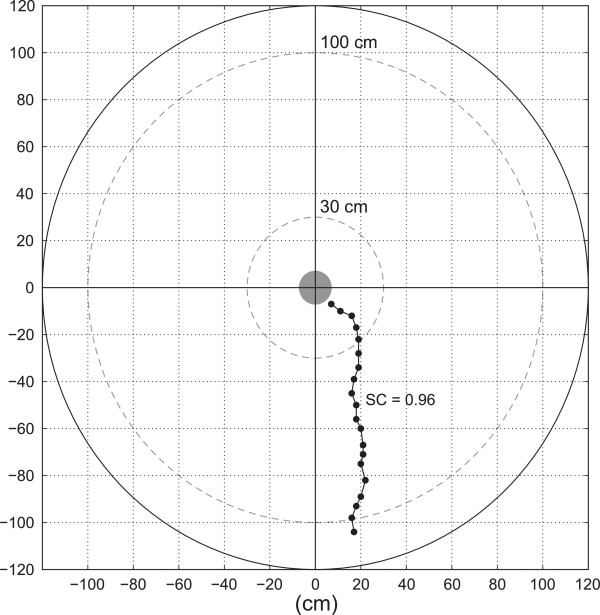
**Arena trajectory.** Example of a single coded trajectory from the arena experiment, using a graphical arena representation as an interface. Solid outer circle represents the arena wall, small filled gray circle represents the release device, black dots connected by an interpolation line represent each hop of the frog in the arena. The orientation bearing was measured at the outer dashed circle (100 cm) and the straightness coefficient (SC) was calculated for the path between inner (30 cm) and outer (100 cm) dashed circles.

In total, we analyzed 38 valid arena trials. We used a custom script written by the first author for MATLAB 7.11.0.584 (The MathWorks Inc., Natick, MA, USA) to code and extract the trajectory of each frog in the arena. A graphical representation of the arena with the reference grid was simultaneously displayed with the video recording of each trial and each hop position of the frog was coded with a mouse click at the corresponding location in the graphical representation. Each position was exported as the corresponding X- and Y-coordinates with the origin at the center of the arena. Arena trajectories were analyzed between an inner circle with 30 cm radius and an outer circle with 100 cm radius, in order to avoid the wall influenced movements close to the release device and close to the arena wall (Figure 4).

We used the coordinates to calculate the orientation bearing of each individual at 100 cm away from the center and the SC of their trajectory from 30 cm to 100 cm away from the center. Similar to the telemetry trials, the SC was calculated as the ratio between the straight line and the actual path distance in the arena. The expected homeward orientation bearings were calculated from the coordinates of each capture position as recoded on the digital map and the center of the arena.

To test for significant homeward orientation in different distance groups, we used the second order Hotelling’s circular test for significant unimodal distribution of each sample. In addition, we looked for significant differences between distributions obtained from displacements over different distances, by using the Hotelling’s two sample test. For significant mean directions a 95% confidence interval was calculated. Statistical analyses were performed with Oriana 4.02.

Our study was approved by the scientific committee of the research station where fieldwork was conducted. All necessary permissions were provided by the ‘Centre National de la Recherche Scientifique’ (CNRS) and by the ‘Direction Régionale de l’Environment de Guyane’ (DIREN). All sampling was conducted in strict accordance with current French and EU law and followed the ASAB guidelines for the treatment of animals in behavioral research and teaching.

## Competing interests

The authors declare that they have no competing interests.

## Authors’ contributions

AP designed the study, collected, analyzed, and interpreted the data and drafted the manuscript. MCL participated in the design of the study, data collection, analysis, and interpretation. LL participated in the statistical analysis and interpretation of the data. MR participated in the design of the study and advised on the GIS data processing. WH participated in the design of the study. MCL, LL, MR and WH reviewed and provided valuable comments on the manuscript. All authors read and approved the final manuscript.
